# Sugary beverage taxation in South Africa: Household expenditure, demand system elasticities, and policy implications

**DOI:** 10.1016/j.ypmed.2017.05.026

**Published:** 2017-12

**Authors:** Nicholas Stacey, Aviva Tugendhaft, Karen Hofman

**Affiliations:** Priority Cost Effective Lessons for Systems Strengthening, MRC Wits Rural Public Health and Health Transitions Unit, School of Public Health, University of the Witwatersrand, 27 St. Andrews Road, Parktown, 2193 Johannesburg, South Africa

**Keywords:** Sugary beverages, Tax, South Africa

## Abstract

South Africa faces a severe and growing obesity epidemic. Obesity and its co-morbidities raise public and private expenditures on healthcare. Sugary beverages are heavily consumed in South Africa and are linked to the onset of overweight and obesity. Excise taxation of sugary beverages has been proposed and adopted in other settings as a means to reduce harms from their consumption. A tax on the sugar content of non-alcoholic beverages has been proposed for implementation in South Africa, however, the public health effects and revenue raising potential of this measure hinges on estimates of the targeted beverages own- and cross-price elasticities. This study applies demand system methods by combining expenditure survey data and sub-national price data to provide the first estimates of price and expenditure elasticities for categories of soft drinks that would be subject to South Africa's proposed sugary beverage tax. The results suggest that demand for these products is sufficiently price-elastic such that a significant reduction in consumption may result from a tax.

## Highlights

• An excise tax on sugary beverages has been proposed in South Africa.
• We fit a censored quadratic almost ideal demand system to estimate beverage price elasticities.
• Demand for carbonated soft drinks is price elastic and responsive to taxation.
• Demand for fruit juices is not price elastic.

## 1 Introduction

Internationally and in South Africa, public health advocates have called for policies to reduce sugar-sweetened beverage consumption in efforts to reduce the burden of obesity and non-communicable diseases. South Africa faces a severe and growing obesity epidemic. Between 2003 and 2012, the prevalence of obesity among women grew from 27.4% to 39.2%, and from 7.5% to 10.6% among men (Shisana et al., 2013). Contemporaneous to this rise in obesity, South Africa has seen a significant rise in the consumption of processed and ultra-processed foods, including sugary beverages (Igumbor et al., 2012). In 1991 the annual per capita consumption of Coca-Cola products was 132 servings, but had risen to 254 by 2010, placing South Africa among the top 10 global consumers of Coca Cola products (Igumbor et al., 2012).

Sugary beverages (SBs) are non-alcoholic beverages containing added or free sugars that the nutrition epidemiology literature links to a number of adverse health conditions (Hu, 2013). The World Health Organization (WHO) defines free sugars to be any sugar added to foods and drinks by a manufacturer, cook or consumer, and any other sugars not in their natural form (World Health Organization, 2015). Thus, sugary beverages include not only carbonated soft drinks but also fruit juices and some dairy products. Meta-analyses of observational studies and randomized trials have associated consumption of SBs to the onset of not only obesity, but also its co-morbidities including type 2 diabetes mellitus (Malik et al., 2013, Malik et al., 2010, Malik et al., 2006). Beyond obesity and metabolic conditions, the high sugar-content of SBs is also linked to increased incidence of dental caries (Bernabe et al., 2014). The chronic nature of the diseases associated with SB consumption places a significant strain on the already resource-constrained HIV/AIDS-laden healthcare system in South Africa.

Recognizing the ramifications of obesity, the National Department of Health's 2015–2020 Strategy for the Prevention and Control of Obesity in South Africa identified various prevention policies including the implementation of a sugar-sweetened beverage tax. Consequently, the 2016/2017 budget announcement stated that as of April 2017 South Africa would implement a tax on sugar-sweetened beverages.

Various countries have adopted forms of sugary beverage taxation, most prominently Mexico, but also Chile, Denmark, France, Hungary and others. In 2014, Mexico implemented a package of nutrition-focused excise taxes, including a 1 peso per liter specific duty on non-alcoholic beverages with added sugar. Although only recently implemented, evaluations of the tax's impact suggest it has been effective in increasing price and deterring purchase. Analyses of routinely collected price data show the tax was almost exactly shifted to retail prices of the targeted beverages, and for carbonated beverages the tax was over-shifted (Colchero et al., 2015b, Gogger, 2015). An empirical evaluation suggests in its first year the tax induced an average 6% reduction in household SSB purchases, as well as a 4% increase in bottled water purchases (Colchero et al., 2016b). The estimated effect increased through 2014 and by the end of the year had induced a 12% reduction in daily per capita SSB purchases (Colchero et al., 2016b). More recent research suggests further reductions in the second year of the tax (Colchero et al., 2016a, Colchero et al., 2017).

Any ex-ante evaluation of the potential effects of such a tax rests on estimates of demand responsiveness to price, otherwise referred to as price elasticity. A price elasticity is a statistic defined as the percentage change in consumption derived from a one-percent change in price (Nghiem et al., 2013). Price elasticities can be own-price elasticities, quantifying the change in demand for a product for a change in that product's price, or alternatively cross-price elasticities quantifying how demand for a product changes when the price of another product changes.

The existing literature on sugary beverage taxes in South Africa is limited to mathematical simulation studies evaluating the health effects of such a tax (Manyema et al., 2015, Manyema et al., 2014). These have drawn on a meta-analysis of studies across multiple countries and settings for elasticity estimates as local estimates were not available (Cabrera Escobar et al., 2013). There is some related literature estimating elasticities for tobacco, alcohol and food products in South Africa, however, at present there are no studies of SB demand elasticities (Agbola, 2015, Alderman and del Ninno, 1999, Case, 1998, Ground and Koch, 2008, Selvanathan and Selvanathan, 2004, Van Walbeek and Blecher, 2015).

This paper seeks to address this gap in the literature. We constructed a novel dataset by combining sub-national price data with the Statistics South Africa Income and Expenditure Survey (IES) 2010/2011. Using this novel dataset we apply standard methods to estimate demand system parameters and to estimate price and expenditure elasticities for a selection of non-alcoholic beverages that would potentially be subject to a tax. The paper proceeds with a description of methods and data, a presentation of the resulting elasticity estimates and a discussion of the policy implications of the results.

## 2 Methods

To estimate price elasticities of relevant products, we adopt an approach based on the Almost Ideal Demand system developed originally by Deaton and others (Banks et al., 1997, Deaton and Muellbauer, 1980a, Deaton and Muellbauer, 1980b). This demand system approach derives relationships between the share of expenditure on a product on its price and the prices of other products and the total value of expenditure being undertaken by households. Based on these relationships, a system of regressions is estimated and elasticities calculated, as described in greater detail in the Appendix.

We combine detailed household-level expenditure data drawn from Statistics South Africa's Income and Expenditure Survey (IES) 2010/2011, and regionally disaggregated average product price data collected by Statistics South Africa's Consumer Price Index (CPI) unit, to estimate a system of regressions of products' expenditure share on all included products' prices, controlling for household characteristics. In addition, the particular approach adopted here accounts for the censoring arising from a significant prevalence of zero expenditures reported by households. As CPI data are largely collected in urban areas, we restrict our IES sample of households to only urban households.

We limit our system to include sugary beverage products potentially subject to the proposed tax, other non-alcoholic beverages, and sugar. The six product categories in the demand system are: (1) carbonated soft drinks, (2) concentrates, (3) fruit juice, (4) tea and coffee, (5) milk, and (6) sugar. While other sugary beverages do exist in the South African setting, including energy drinks, sports drinks, iced teas and others, expenditure and price data were not collected on those items and therefore could not be included in this analysis. However, as those beverages represent a very small share of the soft drink market, their exclusion is not of significant consequence (Euromonitor, 2015). In addition, data limitations prevented us from being able to distinguish regular and diet (artificially sweetened) beverages particularly among carbonated soft drinks (CSD). However, diet beverages take up a very small portion of the non-alcoholic beverage market (Euromonitor, 2015).

To test the robustness of our approach, we estimate various other specifications of the demand system. This includes, testing the utilization of other household variables as controls, combining fruit juice and CSD into a single sweetened soft drink variable, and including junk foods as a part of the demand system. We make the results of these analyses available in supplementary tables. While there is some minor variation in the resulting point estimates, the results are consistent with our main specification presented in this paper.

## 3 Results

### 3.1 Descriptive statistics

Table 1 presents descriptive statistics of the sample of households and their beverage expenditures. As the sample is restricted to urban households it skews from the national population in demographic composition by having a lower proportion of black household heads and a greater proportion of white household heads. Of the beverages that would potentially be subject to a tax under consideration in this study, mean expenditure is greatest on carbonated soft drinks (CSDs). This is consistent with aggregate sales patterns (Euromonitor, 2015).

**Table 1:** Descriptive statistics.

Demographic characteristics	Mean	95% CI	Economic characteristics	Mean	95% CI
Household size	3.512	[3.468, 3.556]	Monthly income	12,587.957	[12,147.53, 13,028.39]
Female household head	0.344	[0.335, 0.353]	Monthly consumption expenditure	9878.861	[9477.671, 10,280.05]
Black household head^a^	0.674	[0.664, 0.684]			
Colored household head	0.115	[0.110, 0.120]			
Asian household head	0.037	[0.033, 0.041]			
White household head	0.173	[0.164, 0.182]			


Product category	Mean	95% CI	Mean	95% CI
	Mean monthly expenditure (2011 ZAR)		Mean price (2011 ZAR/liter)	
Carbonated soft drinks	37.488	[36.111, 38.865]	19.557	[19.532, 19.582]
Concentrates	5.458	[5.017, 5.899]	4.212	[4.201, 4.223]
Fruit juices	14.386	[13.61, 15.162]	13.72	[13.705, 13.735]
Tea & coffee combined	12.977	[12.282, 13.672]	0.731	[0.730, 0.732]
Milk	46.336	[44.839, 47.833]	9.477	[9.469, 9.485]
Sugar	25.454	[24.611, 26.297]	9.121^b^	[9.109, 9.133]

	Prevalence of non-zero expenditure		Mean of non-zero expenditures (2011 ZAR)	
Carbonated soft drinks	0.54	[0.53, 0.55]	69.46	[67.28, 71.64]
Concentrates	0.13	[0.12, 0.14]	40.81	[38.24, 43.38]
Fruit juices	0.25	[0.24, 0.26]	58.25	[55.98, 60.52]
Tea & coffee combined	0.21	[0.2, 0.22]	61.77	[59.5, 64.04]
Milk	0.61	[0.6, 0.62]	75.35	[73.26, 77.44]
Sugar	0.44	[0.43, 0.45]	58.35	[56.88, 59.82]

Notes: IES 2010/2011. N = 13,364 (limited to urban residents only).

a We refer to population group designations standard in South Africa, with Black referring to individuals of African ancestry, Colored of mixed ancestry, Asian of Asian ancestry and White of European ancestry.

b Sugar price is in 2011 ZAR per kilogram.

Taking a closer look at expenditure on CSDs, Table 2 presents details on the distribution of expenditure by socio-economic and demographic household characteristics. First, Table 2 documents the significant prevalence of zero-expenditures. Across the subgroups present, around 50% of households report zero expenditure on CSDs. Expenditure on CSDs is increasing in income, with the highest income quintile spending nearly three times that of the lowest. Across population groups, while white-headed households on average spend more than other population groups, the prevalence of non-zero expenditures is similar across groups indicating the driver of differences is the extent of expenditure among those households who do spend. In addition, we present similar details on household expenditure on fruit juice in Table 3. Mean expenditure is significantly lower than on CSDs, with fewer households report any expenditure on fruit juice. There is a much steeper income gradient in expenditure on fruit juice, with expenditure concentrated among the highest income quintile.

**Table 2:** Carbonated soft drink expenditure by household characteristics.

	Mean expenditure (2011 ZAR)		Prevalence of non-zero expenditure		Mean of non-zero expenditures (2011 ZAR)	
	Mean	95% CI	Mean	95% CI	Mean	95% CI
Income quintile						
1	18.69	[16.72, 20.66]	0.40	[0.38, 0.42]	47.25	[43.10, 51.40]
2	21.52	[19.80, 23.24]	0.44	[0.42, 0.46]	49.37	[46.39, 52.35]
3	27.74	[26.00, 29.48]	0.51	[0.49, 0.53]	54.03	[51.48, 56.58]
4	40.28	[38.07, 42.49]	0.60	[0.58, 0.62]	66.65	[63.63, 69.67]
5	61.79	[57.67, 65.91]	0.64	[0.62, 0.66]	96.18	[90.59, 101.77]

Population group of household head						
Black	30.39	[29.25, 31.53]	0.52	[0.51, 0.53]	58.47	[56.68, 60.26]
Colored	49.62	[46.09, 53.15]	0.59	[0.57, 0.61]	84.43	[79.45, 89.41]
Indian	34.13	[28.17, 40.09]	0.48	[0.42, 0.54]	70.48	[60.89, 80.07]
White	57.71	[51.95, 63.47]	0.60	[0.57, 0.63]	96.66	[88.26, 105.06]

Gender of household head						
Male	42.38	[40.47, 44.29]	0.57	[0.56, 0.58]	74.37	[71.49, 77.25]
Female	28.16	[26.56, 29.76]	0.48	[0.46, 0.50]	58.42	[55.69, 61.15]

Household size						
1–3	33.07	[31.26, 34.88]	0.52	[0.51, 0.53]	63.49	[60.51, 66.47]
4–6	44.47	[42.05, 46.89]	0.58	[0.56, 0.60]	77.00	[73.38, 80.62]
7–9	36.88	[32.46, 41.30]	0.51	[0.47, 0.55]	72.71	[65.63, 79.79]
10 +	38.21	[27.54, 48.88]	0.50	[0.42, 0.58]	75.70	[57.20, 94.20]

Notes: IES 2010/2011. N = 13,364 (limited to urban residents only).

**Table 3:** Fruit juice expenditure by household characteristics.

	Mean expenditure (2011 ZAR)		Prevalence of non-zero expenditure		Mean of non-zero expenditures (2011 ZAR)	
	Mean	95% CI	Mean	95% CI	Mean	95% CI
Income quintile						
1	4.44	[3.69, 5.19]	0.12	[0.1, 0.14]	35.63	[32.02, 39.24]
2	5.11	[4.31, 5.91]	0.14	[0.12, 0.16]	37.28	[33.22, 41.34]
3	8.23	[6.99, 9.47]	0.19	[0.17, 0.21]	42.22	[37.49, 46.95]
4	12.13	[10.95, 13.31]	0.24	[0.22, 0.26]	49.74	[46.4, 53.08]
5	31.68	[29.36, 34]	0.42	[0.4, 0.44]	75.66	[71.59, 79.73]

Population group of household head						
Black	9.5	[8.85, 10.15]	0.2	[0.19, 0.21]	48.07	[45.79, 50.35]
Colored	17.87	[15.47, 20.27]	0.28	[0.26, 0.3]	63.7	[56.55, 70.85]
Indian	16.82	[12.61, 21.03]	0.27	[0.22, 0.32]	61.49	[50.26, 72.72]
White	30.54	[27.52, 33.56]	0.41	[0.38, 0.44]	74.34	[69.06, 79.62]

Gender of household head						
Male	16.17	[15.09, 17.25]	0.26	[0.25, 0.27]	63.35	[60.27, 66.43]
Female	10.99	[10.07, 11.91]	0.23	[0.22, 0.24]	47.52	[44.85, 50.19]

Household size						
1–3	13.1	[12.17, 14.03]	0.24	[0.23, 0.25]	54.39	[51.85, 56.93]
4–6	17.51	[15.95, 19.07]	0.26	[0.24, 0.28]	66.24	[61.72, 70.76]
7–9	10.4	[8.16, 12.64]	0.22	[0.19, 0.25]	47.01	[38.95, 55.07]
10 +	8.91	[5.41, 12.41]	0.2	[0.14, 0.26]	44.55	[36.5, 52.6]
						

Notes: IES 2010/2011. N = 13,364 (limited to urban residents only).

### 3.2 Elasticities

Table 4 presents the resulting matrix of own- and cross-price elasticities, and total expenditure elasticities. Own-price elasticities measure the percent change in the demand for a product when there is a 1% increase in the price of that product, and is shown on the diagonal in Table 4. The estimated own price elasticities: of carbonated soft drinks is − 1.18, of concentrates is − 1.17, of fruit juices and tea & coffee is not statistically significant, of milk is − 1.1 and of sugar is − 2.42. The magnitude of the CSD own-price elasticity is greater than one, implying that CSDs are price elastic with a 20% price increase corresponding to a nearly 24% reduction in demand for carbonated soft drinks.

**Table 4:** Demand system estimates of own-price, cross-price and total expenditure elasticities.

Elasticity	Price						Total expenditure
	CSDs	Concentrates	Fruit juices	Tea & coffee	Milk	Sugar	
CSDs	− 1.18	− 0.59	− 0.91	− 0.97	− 1.28	− 0.78	1.03
	[− 1.62, − 0.74]	[− 0.92, − 0.26]	[− 1.35, − 0.47]	[− 1.43, − 0.51]	[− 1.73, − 0.83]	[− 1.2, − 0.36]	[0.96, 1.1]
Concentrates	1.17	− 1.17	− 0.28	0.17	− 0.98	0.68	0.94
	[0.52, 1.82]	[− 1.93, − 0.41]	[− 1.24, 0.68]	[− 0.82, 1.16]	[− 1.94, − 0.02]	[− 0.14, 1.5]	[0.84, 1.04]
Fruit juices	0.33	0.38	− 0.44	0.86	0.46	0.82	0.97
	[− 0.31, 0.97]	[− 0.26, 1.02]	[− 1.42, 0.54]	[0.05, 1.67]	[− 0.35, 1.27]	[0.06, 1.58]	[0.93, 1.01]
Tea & coffee	0.97	1.4	1.68	0.58	0.68	1.8	1.02
	[0.4, 1.54]	[0.82, 1.98]	[0.87, 2.49]	[− 0.35, 1.51]	[− 0.13, 1.49]	[1.05, 2.55]	[0.93, 1.11]
Milk	− 0.53	− 0.27	− 0.13	− 0.32	− 1.1	0.83	0.97
	[− 0.83, − 0.23]	[− 0.53, − 0.01]	[− 0.53, 0.27]	[− 0.72, 0.08]	[− 1.55, − 0.65]	[0.56, 1.1]	[0.93, 1.01]
Sugar	0.09	0.36	0.39	0.52	1.43	− 2.42	1.25
	[− 0.47, 0.65]	[− 0.07, 0.79]	[− 0.12, 0.9]	[− 0.05, 1.09]	[0.93, 1.93]	[− 3.06, − 1.78]	[1.15, 1.35]

Notes: IES 2010/2011. N = 13,364 (limited to urban residents only). Elasticities estimated via censored quadratic almost ideal demand system estimation. We report mean point estimates with 95% confidence intervals in brackets below.

Cross-price elasticities measure the percent change in the demand for a product when there is a 1% increase in the price of an alternate product, and is shown on the off-diagonals of Table 4. Positive cross-price elasticities suggest that products are substitutes, while negative cross-price elasticities suggest products are complements. Focusing on the CSD and Concentrates columns of Table 4, as the tax would likely impact the price of CSDs and concentrates, the resulting point estimates suggest that an increase in the price of CSDs would induce substitution to carbonates, however, this substitution would be limited as the increase in the price of concentrates would independently reduce their demand. In addition, the elasticity of demand for sugar with respect to CSDs and concentrates is not statistically different from zero.

In addition to price elasticities, we also report total expenditure elasticities. These quantify the change in demand for a product for a specified change in the total budget households have to allocate to expenditure on the product. The total expenditure elasticity of the products studied is positive and approximately unit elastic (value very close to 1) across the products studied indicating proportionate increases in income will result in proportionate increases in demand for the beverages.

## 4 Discussion

Following the National Department of Health's inclusion of a SB tax as an objective in its strategic plan to tackle obesity, the South African National Treasury has proposed the implementation of a SB tax (Treasury, 2016). Given this interest and the subsequent debate around the use of SB taxation, there is a need for evidence on the potential effectiveness of such taxes. To meet this need, we document the patterns of expenditure on non-alcoholic beverages in South Africa and estimate a censored QUAIDS demand system to derive relevant price elasticities of demand.

The magnitude of the estimated elasticities suggests that the proposed tax could act to significantly reduce SSB consumption among urban households in South Africa. With an estimated own-price elasticity of approximately − 1.18, and assuming a complete pass through of the tax to the retail price as observed in other settings, all else equal a 20% tax would induce a 23.6% reduction in consumption of CSDs. The ceteris paribus assumption is necessary as there may be other means industry could adopt to stimulate demand in response to a tax, including advertising and product reformulation. The results are broadly consistent with elasticity estimates from other settings, with the own-price elasticity of sugary beverages found to be − 1.16 in Mexico, − 1.2 in Ecuador, and − 1.23 in a previous meta-analysis of available evidence (Cabrera Escobar et al., 2013, Colchero et al., 2015a, Paraje, 2016).

While the elasticity used in recent modelling studies for South Africa is slightly higher (− 1.29), the implied reduction in sugary beverage consumption of 23% is comparable and likely sufficient to result in energy intake reductions affecting obesity and diabetes (Manyema et al., 2014, Manyema et al., 2015, Manyema et al., 2016). However, while the CSD own-price elasticity being greater than one suggests the potential for public health benefits from taxation, the revenue raising potential of their taxation is limited – tax increases might result in decreasing gains in revenue if fully passed through to the price.

At the time of writing, South African policymakers have considered various rates and designs for the tax. Initially, a 0.0229 ZAR/g of sugar rate was proposed for all non-alcoholic drinks containing added sugar, an approximate 20% tax (Treasury, 2016). Subsequently, following industry lobbying this has been lowered to a 10% tax, with an exemption for the first four grams of sugar. What structure ultimately to be adopted is at present uncertain. The stated goal of levying the tax based on sugar content is to incentivize reformulation among producers (Treasury, 2016). The gains to producers from reformulating are a function of the magnitude of the demand price elasticity they face. If demand for their product is highly price sensitive reducing the extent to which they have to increase their product's price will protect their sales, and thus producers will have greater incentive to reformulate. As the decision to reformulate will also be driven by the un-observed costs of doing so, it is difficult to anticipate ex-ante the likelihood it will occur. Should reformulation occur price-induced reductions in beverage intake would be smaller, but the reduced sugar content of the products would reduce caloric intake and provide health benefits.

While the distributional incidence of individual tax policies should be viewed in the context of the broader fiscal system of income taxation, service provision and transfers, concerns are often raised that SB taxes might be regressive – i.e., disproportionately affecting lower-income persons. However, Table 2 demonstrates that there is a strong positive relationship between CSD expenditure and income, and similarly Table 4 presents evidence of a positive total expenditure elasticity. Since consumption of SB is less likely among lower-income persons and rises with income, concerns about tax regressivity might not be fully applicable. Due to data limitations we are not able to stratify our sample and estimate price elasticities by income group, but there is growing evidence that the poor are more price-responsive, which further reduces tax regressivity concerns (Colchero et al., 2016b, van Walbeek, 2002). Nevertheless, South Africa has a highly progressive fiscal system, with progressive income taxation, subsidized public services and significant transfers to the poor (Inchauste et al., 2015).

Unlike other products, fruit juice is estimated to have a relatively small and not statistically significant own-price elasticity. There has been some debate as to whether fruit juices should be subject to the tax or not, with arguments against the tax focusing on the products' relative nutrient value and contributions to rural employment and incomes. The arguments for the tax are based on a growing literature showing that the effects of fruit juice on weight gain and diabetes are comparable to those of other sugary beverages (Muraki et al., 2013, Odegaard et al., 2010, Pase et al., 2015, Popkin and Hawkes, 2016, Shefferly et al., 2016). The negligible elasticity suggests that public health arguments for their inclusion may currently be misplaced and that the tax will not yield meaningful reductions in sugar intake from their consumption at this time, and by the same token that arguments regarding the potential economic harm caused by their taxation may be overstated. The implications of our finding of a weak link between juice prices and consumption is could change if the SB tax induced the food industry to promote fruit juice as an alternative to other sugary beverages.

The study is subject to a number of limitations. First, the price data used here do not include all non-alcoholic beverages categories, particularly mineral/bottled water, sports and energy drinks, and dairy blends, and as such these products have been excluded from the analysis. Another data related limitation is the inherited definition of carbonated soft drinks. This categorization arises in both the IES and CPI price data and does not adequately distinguish between regular CSDs (subject to the proposed tax) and artificially sweetened, diet CSDs (not subject to the proposed tax), and whose consumption has differing effects on obesity. Euromonitor sales data suggest that this is likely not of significance, as diet drinks in 2010/2011 constituted only 4% of national CSD sales (Euromonitor, 2015).

## 5 Conclusions

South Africa's growing obesity epidemic poses a significant burden to the public health system and the economic well-being of households. Soft drinks that are high in sugar are heavily consumed in South Africa and are linked to the onset of obesity, and thus have been targeted by policymakers for taxation. This study combines CPI product price data with expenditure data from the Income and Expenditure Survey to provide the first price elasticity estimates of soft drinks in South Africa. Carbonated soft drinks have the highest expenditure among soft-drink categories. The elasticities estimated here suggest that the proposed tax on SBs is likely to have a significant public health impact.

## Conflict of interest

The authors declare there is no conflict of interest.

## Funding

This work was supported by the University of North Carolina and The Bill and Melinda Gates Foundation, Seattle, WA [Grant number #OPP1098574] through The Centre for Disease Dynamics, Economics & Policy, Washington, D.C.

## Appendix A Appendix: data and methods

### A.1 Datasets

This study estimates a demand system using the Statistics South Africa Income and Expenditure Survey (IES) of 2010/2011. The IES is a nationally representative household survey conducted periodically by Statistics South Africa in part to ascertain patterns of expenditure to inform the Consumer Price Index (CPI) basket definition. It collects disaggregated expenditure data at the household level, including expenditure on various sub-categories of non-alcoholic beverages. Expenditure on these items was collected by diary method, with all adults residing in a participating household documenting their expenditure for two consecutive two-week periods.

Expenditure surveys typically include a measure of the quantity of each product purchased, as well as expenditure on each product or product category. This allows for the estimation of price implicitly as expenditure per unit, or in other settings the price paid is recorded explicitly. However, the IES only contains the value of expenditure on goods at the product or Classification of Individual Consumption According to Purpose (COICOP) code level and does not include price or quantity information. Consequently, this study draws on province-level time series product price data collected by the Statistics South Africa Consumer Price Index unit. For each household, prices are merged to the expenditure data based on COICOP codes and the household's province and month of survey in the IES. As the collection of price data is restricted to urban areas, we limit our analytical sample to only urban households.

The Statistics South Africa Consumer Price Index prices are reported across various package sizes for each beverage product and therefore are standardized to a single per liter price by constructing the geometric mean of the effective per liter price across all the available package size prices. In addition, certain beverage classes like concentrates and infusions are not typically purchased in ready-to-drink form, and as such for these products we calculate the per liter price after applying reconstitution factors. This ensures that all beverage prices are standardized to ready-to-drink versions per liter.

### A.2 Demand system estimation

Estimation of demand systems begins with specifying a generic functional form for households' indirect utility/satisfaction (or equivalently expenditure function), and applying standard results from microeconomic theory to derive a system of Engel curves for the products purchased by households (Deaton and Muellbauer, 1980a, Deaton and Muellbauer, 1980b). The parameters of the derived Engel curves are estimated by maximum likelihood, which in turn allow for the estimation of price elasticities. We estimate a version of the flexible quadratic almost ideal demand system (QUAIDS) of Banks, Blundell and Lewbel (Banks et al., 1997) that accounts for censoring (due to non-negligible proportion of non-expenditure on certain product categories).

Suppose households allocate their expenditure over *k* product categories, face a vector of 1 × *k* vector of prices, *p*, and have income *m*. By applying Roy's Identity, to a generalized indirect utility function one can derive a system of *k* Engel curve regressions of expenditure share of each product *i*, *w*_*i*_, on prices and total expenditure. To account for the significant presence of non-expenditure on beverages and resulting censoring, we adopt the two-step approach of Shonkwiler and Yen (Shonkwiler and Yen, 1999). The first step is to estimate a probit regression of the probability of non-zero purchase on household characteristics for each product. The second step is to estimate an altered version of the conventional QUAIDS system, taking the first step estimates and adjusting the traditional QUAIDS form using estimates of the values of a standard normal cumulative density function (CDF) and probability density function (PDF) as below:

$$w_{\mathrm{ih}}=\left(\alpha_{i}+\sum_{j=1}^{k}\gamma_{\mathrm{ij}}\ln p_{\mathrm{jh}}+\beta_{i}\ln\left\{\;\frac{m_{h}}{a\left(p_{h}\right)}\;\right\}+\frac{\lambda_{i}}{b\left(p_{h}\right)}\;{\left[\;\ln\left\{\;\frac{m_{h}}{a\left(p_{h}\right)}\;\right\}\;\right]}^{\;2}\right).\Phi \left(z_{h}^{\prime }\nu_{i}\right)+\mu_{i}.\phi \left(z_{h}^{\prime }\nu_{i}\right)+{z_{h}^{\prime }\theta +\epsilon }_{\mathrm{ih}}$$

where *h* indexes the observed households, *p*_*ih*_ is the price of product *i*  faced by household *h*, ***p***_*h*_ is the vector of prices faced by household *h*, *m*_*h*_ is total recorded expenditure by household *h*, *z*_*h*_ is a vector of household characteristics, ***ν***_***i***_ are probit coefficient estimates of household characteristics on an indicator of non-zero expenditure on product *i* from the first step, *Φ*(.) is the standard normal CDF, *ϕ*(.) is the standard normal PDF, and *ϵ*_*ih*_ is a household specific mean zero disturbance term. The control variables we include in the vector of household characteristics include household size and seasonality indicators.

The uncompensated price elasticity of product *i* with respect to the price of product *j* for household *h* is given by^1^:

$$\sigma_{\mathrm{ijh}}=-{\;\delta }_{\mathrm{ij}\;}+\frac{1}{w_{\mathrm{ih}}}\left(\gamma_{\mathrm{ij}}-\mu_{\mathrm{imh}}\left[\;\alpha_{j}+\sum_{k}\gamma_{\mathrm{jk}}\ln p_{k}\right]-\frac{\lambda_{i}\beta_{j}}{b\left(p_{h}\right)}{\left[\ln\left\{\frac{m_{h}}{a\left(p_{h}\right)}\right\}\right]}^{2}\right)$$

where:

$$\mu_{\mathrm{imh}}=\beta_{i}+\frac{2{\;\lambda }_{i}}{b\left(p_{h}\right)}\;\ln\left\{\;\frac{m_{h}}{a\left(p_{h}\right)}\;\right\}.$$

and *δ*_*ij*_ is the Kronecker delta. We report mean elasticities for the whole sample, with 95% confidence intervals calculated by way of the delta method.

A long-identified concern with the estimation of demand systems using household-level data is for the potential for price-endogeneity. This typically arises as a measurement-error issue when authors construct “unit-value” prices by dividing expenditure by quantity. If product quality is un-observed as is typically the case, expenditure share and price will be endogenous. We adopt the solution proposed by Deaton and adopted by others estimating sugary beverage price elasticities, whereby we use regional average product prices (Colchero et al., 2015a, Deaton, 1987). This is in part a direct outcome of the available price data. This limits the extent to which local supply shocks influence purchase decisions, and the extent to which quantity-quality decisions at the household level could introduce endogeneity between product prices and household expenditure shares. Other solutions to these issues involve the use of instrumental variables (Zhen et al., 2014).

## Appendix B Supplementary data

Supplementary tables Image 1
