# Supplementary material for: Sugary beverage taxation in South Africa: Household expenditure, demand system elasticities, and policy implications
Source: Prev Med. 2017 Dec;105(Suppl):S26–31. doi: 10.1016/j.ypmed.2017.05.026 (PMC5747348; doi:10.1016/j.ypmed.2017.05.026)
Supplement: Supplementary file 1 — Supplementary tables [file mmc1.docx]

**Supplementary Tables**

1. Demand system estimates of own-price, cross-price and total expenditure elasticities combining CSDs and Fruit juices Elasticities from system including junk snack foods (chocolates and crips)
2. Demand system estimates of own-price, cross-price and total expenditure elasticities including junk snack foods (chocolates and crips)
3. Demand system estimates of own-price, cross-price and total expenditure elasticities controlling for educational attainment (high school completion) of household head

Supplementary Table 1: Demand system estimates of own-price, cross-price and total expenditure elasticities combining CSDs and Fruit juices

| Elasticity | Price | | | | | Total Expenditure |
| --- | --- | --- | --- | --- | --- | --- |
|  | CSDs & FJ | Concentrates | Tea & Coffee | Milk | Sugar |  |
| CSDs & FJ | -1.51 | 0.53 | -0.37 | -0.12 | -0.04 | 0.93 |
|  | [-1.92, -1.1] | [0.27, 0.79] | [-0.73, -0.01] | [-0.36, 0.12] | [-0.49, 0.41] | [0.88, 0.98] |
| Concentrates | 1.15 | -1.01 | -0.91 | -0.25 | 1.2 | 0.79 |
|  | [0.75, 1.55] | [-1.4, -0.62] | [-1.33, -0.49] | [-0.58, 0.08] | [0.64, 1.76] | [0.72, 0.86] |
| Tea & Coffee | 0.67 | 0.14 | 0.18 | 0.82 | 1.15 | 1.09 |
|  | [0.29, 1.05] | [-0.23, 0.51] | [-0.33, 0.69] | [0.56, 1.08] | [0.82, 1.48] | [0.99, 1.19] |
| Milk | 0.08 | -0.93 | -0.4 | -0.74 | 1.29 | 0.81 |
|  | [-0.5, 0.66] | [-1.4, -0.46] | [-0.95, 0.15] | [-1.19, -0.29] | [0.38, 2.2] | [0.67, 0.95] |
| Sugar | -2 | -0.66 | 0.25 | -1.07 | -5.86 | 1.24 |
|  | [-2.65, -1.35] | [-1.17, -0.15] | [-0.32, 0.82] | [-1.57, -0.57] | [-7.74, -3.98] | [1.14, 1.34] |
| Notes: IES 2010/2011. N = 13364 (limited to urban residents only). Elasticities estimated via censored quadratic almost ideal demand system estimation. We report mean point estimates with 95% confidence intervals in brackets below. | | | | | | |

Supplementary Table 2: Demand system estimates of own-price, cross-price and total expenditure elasticities including junk snack foods (chocolates and crips)

| Elasticity | Price | | | | | | Total Expenditure |  |
| --- | --- | --- | --- | --- | --- | --- | --- | --- |
|  | CSDs | Concentrates | Fruit Juices | Tea & Coffee | Milk | Junk Foods |  |  |
| CSDs | -1.57 | -0.51 | -1.28 | -0.7 | -0.26 | -0.69 | 1 |  |
|  | [-2.04, -1.1] | [-0.84, -0.18] | [-1.78, -0.78] | [-1.15, -0.25] | [-0.7, 0.18] | [-1.05, -0.33] | [0.9, 1.1] |  |
| Concentrates | -0.23 | -2.68 | -2.11 | -2.09 | 0.36 | -1.53 | 0.73 |  |
|  | [-0.93, 0.47] | [-3.3, -2.06] | [-3.29, -0.93] | [-3.06, -1.12] | [-0.77, 1.49] | [-2.28, -0.78] | [0.52, 0.94] |  |
| Fruit Juices | 0.09 | 1.08 | 0.59 | 1.76 | 2.79 | 1.43 | 0.95 |  |
|  | [-0.47, 0.65] | [0.59, 1.57] | [-0.48, 1.66] | [0.99, 2.53] | [1.91, 3.67] | [0.86, 2] | [0.84, 1.06] |  |
| Tea & Coffee | 0.78 | 0.32 | 1.1 | 0.2 | 0.88 | 0.29 | 0.99 |  |
|  | [0.12, 1.44] | [-0.23, 0.87] | [0.22, 1.98] | [-0.78, 1.18] | [0.03, 1.73] | [-0.36, 0.94] | [0.92, 1.06] |  |
| Milk | 0.29 | 0.33 | 0.61 | 0.14 | -2.25 | 0.22 | 1.19 |  |
|  | [-0.16, 0.74] | [00, 0.66] | [0.15, 1.07] | [-0.28, 0.56] | [-2.92, -1.58] | [-0.13, 0.57] | [1.08, 1.3] |  |
| Junk Foods | -0.2 | -0.42 | -0.14 | -1 | 0.95 | -0.61 | 0.97 |  |
|  | [-0.96, 0.56] | [-1.03, 0.19] | [-1.2, 0.92] | [-2.11, 0.11] | [-0.39, 2.29] | [-1.31, 0.09] | [0.88, 1.06] |  |
| Notes: IES 2010/2011. N = 13364 (limited to urban residents only). Elasticities estimated via censored quadratic almost ideal demand system estimation. We report mean point estimates with 95% confidence intervals in brackets below. | | | | | | | | |
|  | | | | | | | | |

Supplementary Table 3: Demand system estimates of own-price, cross-price and total expenditure elasticities controlling for educational attainment (high school completion) of household head

| Elasticity | Price | | | | | | Total Expenditure |  |
| --- | --- | --- | --- | --- | --- | --- | --- | --- |
|  | CSDs | Concentrates | Fruit Juices | Tea & Coffee | Milk | Sugar |  |  |
| CSDs | -1.19 | -0.57 | -0.95 | -0.98 | -1.32 | -0.69 | 1.02 |  |
|  | [-1.63, -0.75] | [-0.9, -0.24] | [-1.4, -0.5] | [-1.45, -0.51] | [-1.79, -0.85] | [-1.09, -0.29] | [0.95, 1.09] |  |
| Concentrates | 1.1 | -1.23 | -0.36 | 0.02 | -1.24 | 0.64 | 0.93 |  |
|  | [0.45, 1.75] | [-1.97, -0.49] | [-1.35, 0.63] | [-0.98, 1.02] | [-2.23, -0.25] | [-0.14, 1.42] | [0.83, 1.03] |  |
| Fruit Juices | 0.45 | 0.66 | -0.16 | 1.19 | 0.79 | 1.06 | 0.98 |  |
|  | [-0.2, 1.1] | [0.06, 1.26] | [-1.17, 0.85] | [0.39, 1.99] | [-0.03, 1.61] | [0.41, 1.71] | [0.93, 1.03] |  |
| Tea & Coffee | 0.92 | 1.37 | 1.73 | 0.56 | 0.85 | 1.67 | 1.01 |  |
|  | [0.36, 1.48] | [0.8, 1.94] | [0.88, 2.58] | [-0.38, 1.5] | [0.01, 1.69] | [0.96, 2.38] | [0.92, 1.1] |  |
| Milk | -0.48 | -0.2 | -0.01 | -0.16 | -0.98 | 0.88 | 0.96 |  |
|  | [-0.78, -0.18] | [-0.45, 0.05] | [-0.41, 0.39] | [-0.55, 0.23] | [-1.44, -0.52] | [0.62, 1.14] | [0.92, 1] |  |
| Sugar | 0.03 | 0.15 | 0.14 | 0.21 | 1.16 | -2.64 | 1.27 |  |
|  | [-0.53, 0.59] | [-0.25, 0.55] | [-0.38, 0.66] | [-0.34, 0.76] | [0.67, 1.65] | [-3.19, -2.09] | [1.17, 1.37] |  |
| Notes: IES 2010/2011. N = 13364 (limited to urban residents only). Elasticities estimated via censored quadratic almost ideal demand system estimation. We report mean point estimates with 95% confidence intervals in brackets below. | | | | | | | | |
|  | | | | | | | | |
